# Ectopic Ureteral Orifice: An Unusual Cause of Urinary Incontinence in an Adult

**DOI:** 10.7759/cureus.116618

**Published:** 2026-09-20

**Authors:** Brahima Kirakoya, Yannick Jean Rodrigue Pingdwendé Traoré, Bienvenue Désiré Ky, Clotaire Alexis Marie Kiemdiba Donega Yaméogo, Fasnewinde Aristide Kaboré

**Affiliations:** 1 Urology, Centre Hospitalier Universitaire Yalgado Ouédraogo, Ouagadougou, BFA

**Keywords:** duplex collecting system, ectopic ureter, ureteral reimplantation, urinary incontinence, vaginal ectopic ureter

## Abstract

Vaginal insertion of an ectopic ureter is a rare anomaly causing continuous incontinence despite normal voiding. We report a 30-year-old woman with lifelong urinary leakage that remained unrecognized until adulthood. Computed tomography urography revealed a left duplex collecting system with an ectopic ureter draining the upper renal pole into the vagina. She underwent successful ureterovesical reimplantation using the Politano-Leadbetter technique, achieving immediate and complete continence. This case emphasizes the need for meticulous evaluation in adults with chronic incontinence to allow for definitive surgical correction.

## Introduction

An ectopic ureter is defined by a ureteral orifice located outside the posterolateral area of the bladder trigone [1]. It is frequently associated with duplication of the collecting system and predominantly affects females [2]. When the orifice opens distal to the bladder sphincter or into the vagina, the clinical picture is characteristic, consisting of permanent urinary incontinence despite a preserved voiding pattern. Diagnosis is often made in childhood; however, some forms may remain unrecognized until adulthood, exposing patients to a prolonged impairment of quality of life.

We report a case of an ectopic ureter opening into the vagina in an adult woman presenting with chronic urinary incontinence.

## Case presentation

A 30-year-old woman presented with permanent urinary incontinence that had been present since childhood. This urine leakage had prompted several consultations since pediatric age. The urinary leakage was both nocturnal and diurnal.

On physical examination, the patient was wearing a pad completely soaked with urine. A pinpoint orifice located below the urethral meatus, slightly lateralized to the left, was continuously leaking urine. The remainder of the physical examination was normal. There was no palpable distended bladder. The neuro-urological examination was normal.

Renal function was normal. Urine culture did not reveal any organisms. Computed tomography urography revealed a duplex excretory system on the left side (Figure 1).

**Figure 1:**
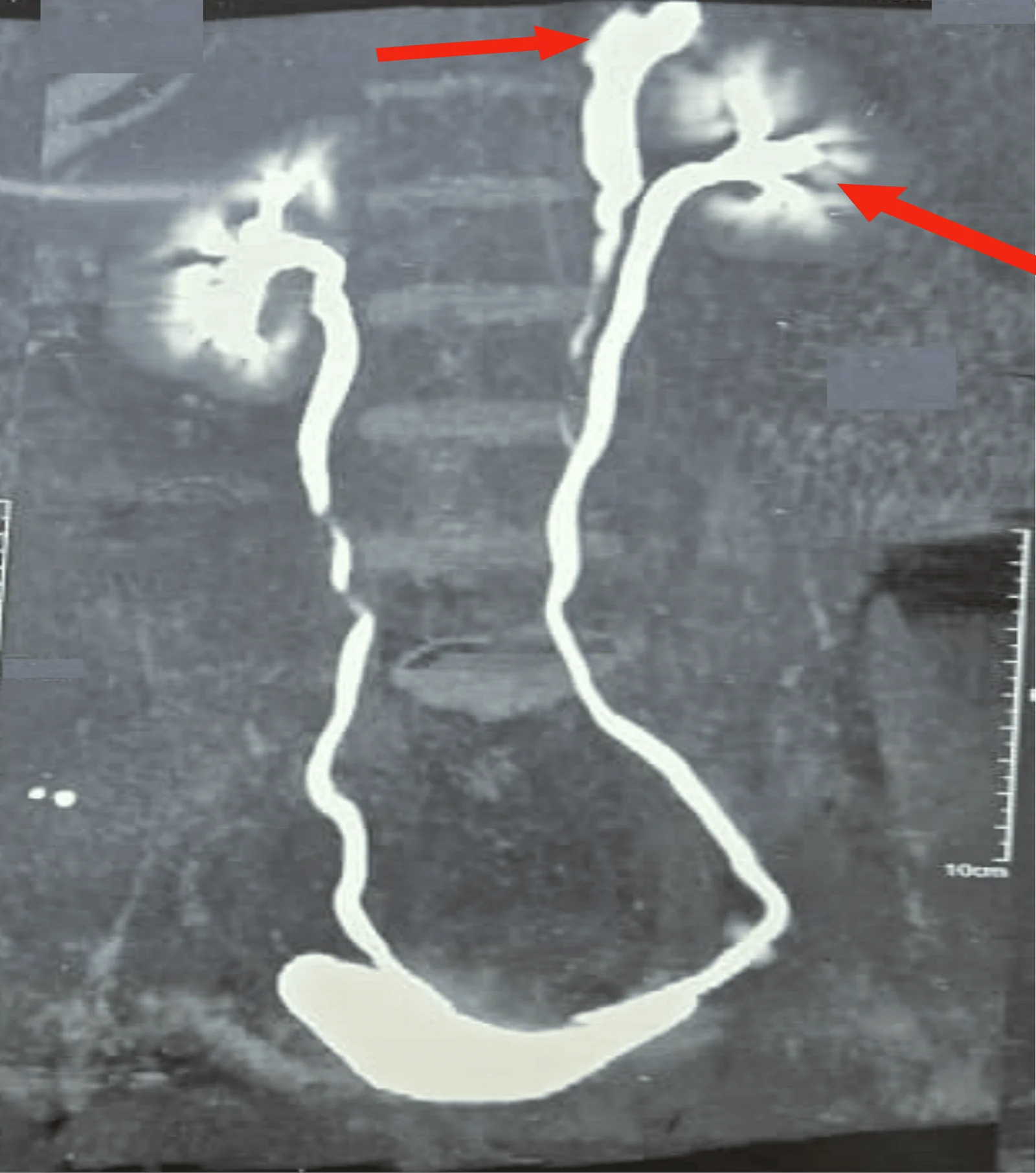
Computed tomography urography showing a left duplex excretory system The red arrows indicate the duplication of the left collecting system.

We catheterized the new orifice with a 6 Fr Nelaton catheter and injected iodinated contrast material. This confirmed the ectopic ureteral drainage of the upper pole of the left kidney (Figure 2). Cystoscopy identified two ureteral orifices, right and left.

**Figure 2:**
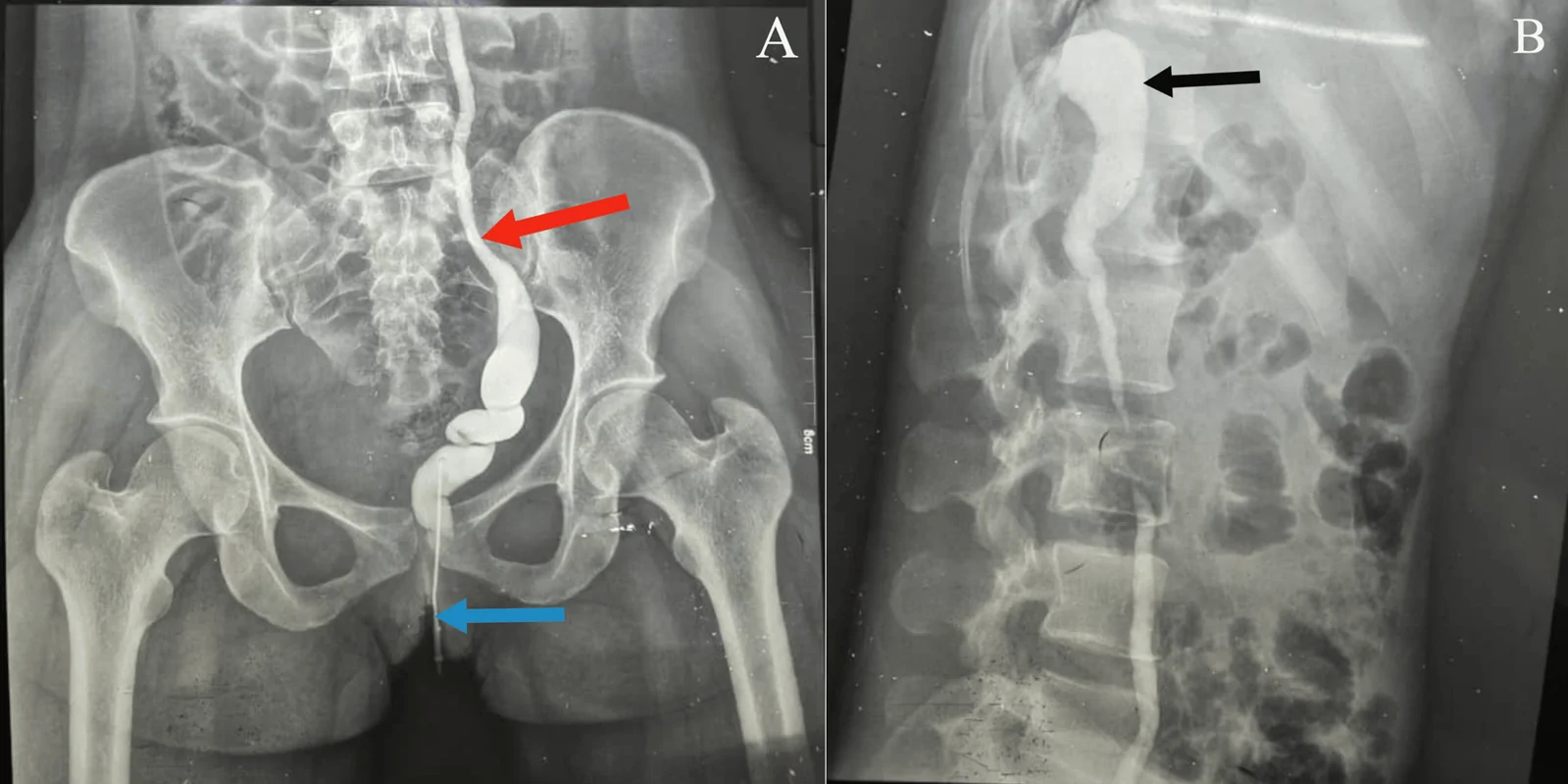
Retrograde ureterography showing the ectopic ureter draining the left upper renal pole (A) Frontal view showing the Nelaton catheter in the ectopic orifice (blue arrow) and the tortuous ectopic ureter (red arrow). (B) View showing the contrast material reaching the upper pole of the left kidney (black arrow).

The patient underwent surgery. Intraoperatively, we identified both ureters. A cystotomy allowed catheterization of the ureter with a normal vesical insertion, which enabled it to be distinguished from the ureter with the ectopic insertion (Figure 3).

**Figure 3:**
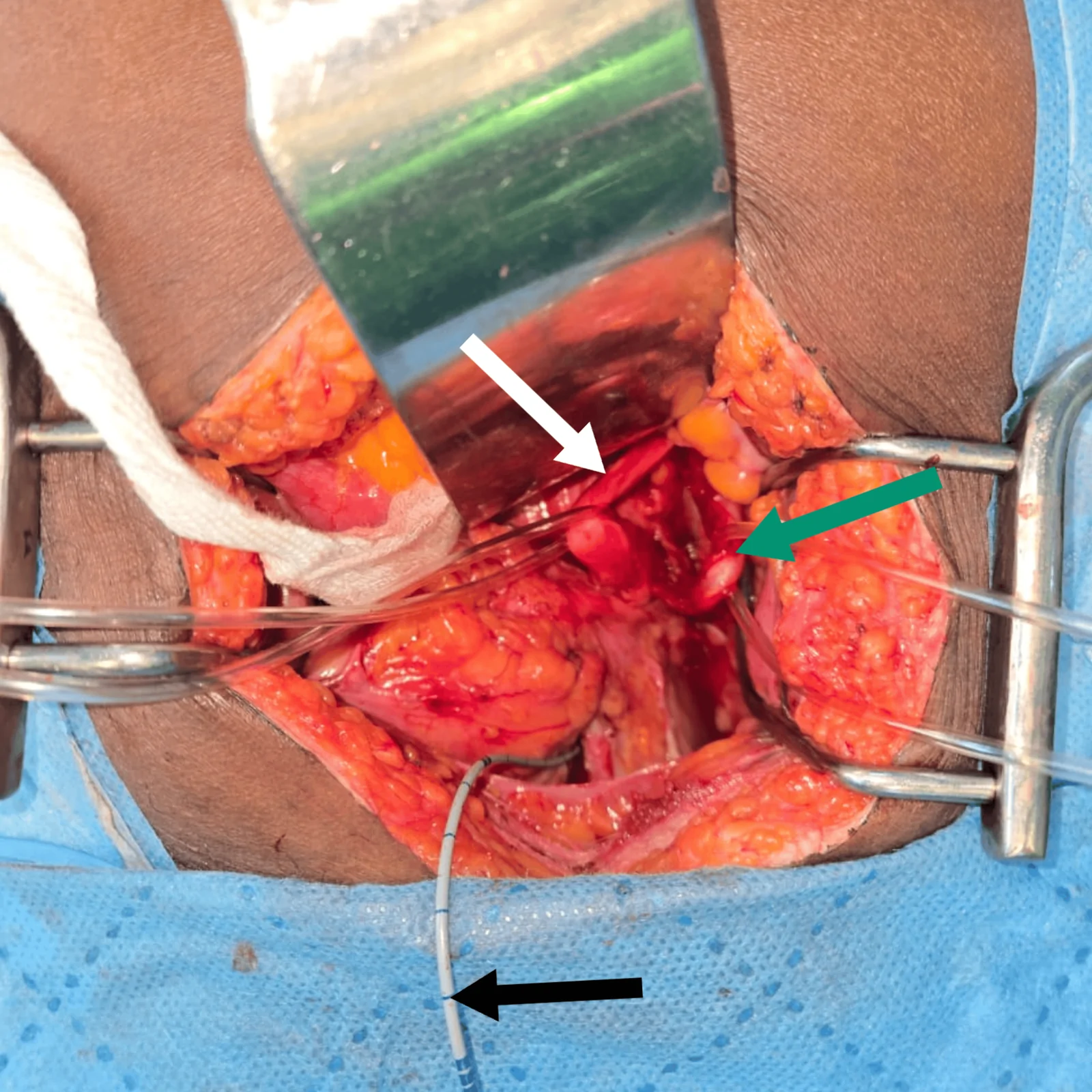
Intraoperative view showing the ectopic and normal left ureters The white arrow indicates the mobilized ectopic ureter. The green arrow points to the normal left ureter. The black arrow highlights the catheter placed into the normal ureter via cystotomy to facilitate their distinction.

We then mobilized the ectopic ureter and performed a Politano-Leadbetter ureterovesical reimplantation over a double-J stent. The postoperative course was uneventful. Urinary leakage ceased immediately. The patient had normal voiding after removal of the urinary catheter on postoperative day 8.

## Discussion

An ectopic ureter is a rare congenital anomaly with an estimated incidence between 1/2000 and 1/4000 live births. It is frequently associated with duplication of the collecting system, as was the case in our observation [3]. In the pediatric population, female predominance is marked, with a ratio reaching up to 6:1. This trend is also observed in the adult population. Among 10 cases of ectopic ureteral insertion in adults collected over 10 years by Toia et al., 9 were women [4]. Clinical presentation also differs by sex, related to the usual insertion sites. In men, ectopic insertion frequently manifests with pain related to the urinary tract. In women, the ectopic orifice is generally located distal to the bladder sphincter, which explains the characteristic presentation of permanent urinary incontinence despite normal voiding [2,4]. The most frequent sites of ectopic insertion in women are the vagina, vulvar vestibule, distal urethra, and uterus [3-5]. The vaginal insertion in the reported case is the cause of the incontinence. The failure to recognize the cause of this incontinence was due to an insufficiently thorough clinical examination, despite the characteristic clinical picture presented by this patient, who had been consulting since childhood. In the study by Toia et al., some patients even underwent placement of a suburethral sling to correct a urinary leak that was not of urethral origin [4].

Diagnostic certainty relies on imaging. Computed tomography urography is considered the reference examination for visualizing the duplication, ureteral course, and site of insertion. MR urography is an interesting alternative, particularly for functional and morphological assessment without irradiation [6,7]. Renal scintigraphy can also be useful to evaluate upper pole function in duplications [8]. Retrograde ureteropyelography can help delineate the course of the ectopic ureter, as was the case in our observation. The ectopic ureter draining the upper pole confirms the Weigert-Meyer law [9]. Often overlooked, the workup should include cystography to look for an associated ipsilateral vesicoureteral reflux.

Management of incontinence due to ectopic ureteral insertion is surgical. The goal is to eliminate the source of the urine leak, either by removing it or by redirecting it into the bladder [10]. When a duplex renal collecting system is involved, the functionality of the moiety whose content is externalized is an important factor in choosing the operative technique. Options include ureterovesical reimplantation, ureteroureterostomy, or hemi-nephroureterectomy [8,10,11]. In our case, since the upper pole of the left kidney was functional, we opted for ureterovesical reimplantation because of the dilation and thickening of the ectopic ureter.

## Conclusions

An ectopic ureter must be considered in the differential diagnosis of adult continuous urinary incontinence, especially when symptoms have persisted since childhood despite normal voiding habits. A meticulous physical examination remains paramount to avoid decades of misdiagnosis and unnecessarily prolonged impairment of quality of life. Furthermore, comprehensive anatomical mapping using cross-sectional imaging is essential for accurate surgical planning. Ultimately, surgical reimplantation yields excellent functional outcomes, highlighting the critical need for prompt urological evaluation when this congenital anomaly is suspected.
